# Retention and mitigation of metals in sediment, soil, water, and plant of a newly constructed root-channel wetland (China) from slightly polluted source water

**DOI:** 10.1186/2193-1801-3-326

**Published:** 2014-06-28

**Authors:** Baoling Wang, Yu Wang, Weidong Wang

**Affiliations:** State Key Laboratory of Environmental Aquatic Chemistry, Research Center for Eco-Environmental Sciences, The Chinese Academy of Sciences, Beijing, 100085 P. R. China; University of Chinese Academy of Sciences, Beijing, 100049 P. R. China

**Keywords:** Pond-wetland complexes, Root-channel zone, Plant-bed/ditch system, Heavy metal, Enrichment factor, Potential ecological risk, Mass balance

## Abstract

Constructed root-channel wetland (CRCW) is a term for pre-pond/wetland/post-pond complexes, where the wetland includes plant-bed/ditch landscape and root-channel structure. Source water out of pre-ponds flows through alternate small ditches and plant beds with root-channels via a big ditch under hydraulic regulation. Then source water flows into post-ponds to finish final polishing. This article aims to explore the potential of components of a pilot CRCW in China on mitigating metals in micro-polluted source water during its initial operation stage. We investigated six heavy metals (Cd, Cr, Cu, Ni, Zn, and Pb) in surface sediment, plant-bed subsurface soil, water, and aquatic plants during 2012–2013. Monitoring results showed that pond/ditch sediments and plant-bed soil retained a significant amount of Cr, Ni, and Zn with 93.1%, 72.4%, and 57.5% samples showing contamination factor above limit 1 respectively. Remarkably the high values of metal enrichment factor (*EF*) occurred in root-channel zones. Water monitoring results indicated that Ni, Zn, and Pb were removed by 78.5% (66.7%), 57.6% (59.6%), and 26.0% (7.5%) in east (west) wetland respectively. Mass balance estimation revealed that heavy metal mass in the pond/ditch sediments accounted for 63.30% and that in plant-bed soil 36.67%, while plant uptake occupied only 0.03%. The heavy metal accretion flux in sediments was 0.41 - 211.08 μg · cm^-2^ · a^-1^, less than that in plant-bed soil (0.73 - 543.94 μg · cm^-2^ · a^-1^). The 1.83 ha wetland has retained about 86.18 kg total heavy metals within 494 days after operation. This pilot case study proves that constructed root-channel wetland can reduce the potential ecological risk of purified raw water and provide a new and effective method for the removal of heavy metals from drinking water sources.

**Electronic supplementary material:**

The online version of this article (doi:10.1186/2193-1801-3-326) contains supplementary material, which is available to authorized users.

## Introduction

Metals, especially heavy metals, have received considerable attention owing to their contamination on aquatic environments. In particular, they are common pollutants in water sources and drinking water, prompting concern regarding their hazardous effects on the environment and the food chain (Graney and Eeriksen 2004; Stead-Dexter and Ward 2004). Moreover, they are known to become concentrated, and diffuse spatially and have been shown to induce adverse effects at physical and biochemical levels, including toxication, carcinogenesis, teratogenesis, and mutagenesis (Weis and Weis 2004; Wu et al. 2013). Many novel technologies and approaches to the treatment of metals have been studied intensively (Rai 2012; Rana et al. 2013). Application of constructed wetland in water treatment has gained popularity throughout the world for its low costs, low energy consumption and minimal operation and maintenance. Constructed wetland can effectively remove heavy metals in water sources and waste water by acting as a matrix, facilitating interaction between microbes and plant/animal communities and performing functions such as filtration, adsorption, precipitation, ionic exchange, microbiological degradation, and biological uptake (Kadlec 2003; Vymazal 2011; Si et al. 2011; Malaviya and Singh 2012). Recent reports have focused primarily on the sources and distributions of heavy metals (Zhang et al. 2002; Protano et al. 2013; Mohammed and Abdu 2013) and on assessing the risks posed by such metals (Xiao et al. 2012; Yang et al. 2008; Vymazal and Krása 2003; Obarska-Pempkowiak and Klimkowska 1999; Goulet et al. 2001).

**Figure 1:**
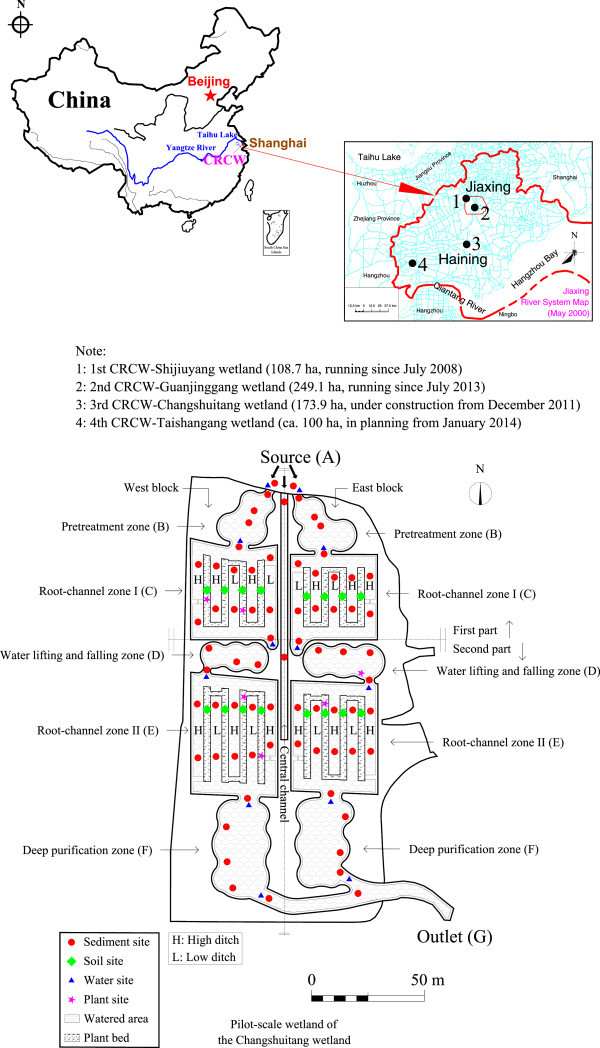
**Location map of constructed root-channel wetlands (CRCWs) and sampling sites in the pilot wetland.** CRCWs are located in the center of Yangtze River delta on China’s southeast coast (top left), and CRCWs 1, 2 in Jiaxing City, CRCWs 3, 4 in Haining City (top right). Sediments from ponds and ditches, soils from plant beds, water, and plant above ground portion were sampled in a pilot-scale wetland of CRCW 3 (down).

The root-channel wetland here studied is also called constructed root-channel wetland (CRCW), a type of constructed wetland proposed by our research team and currently under patent protection. It is an innovative wetland configuration that includes the macroscopic plant-bed/ditch landscapes and mesoscopic root-channel structures originated from Baiyangdian Lake in North China (Wang et al. 2012a; Wang et al. 2012b). Fundamentally, the CRCW technology utilizes various crop stalks (fresh or dry, thick or thin) as substrates that are buried in the wetland subsurface soil layers during the initial operation stages to form artificial root-channels. Simultaneously, aquatic plants with developed rhizomes and adventitious roots are transplanted gradually to form natural root-channels, which allow the efficient transmission of gases, liquids, and other materials into the deep soil matrix. A successful CRCW case study has been undertaken in the Shijiuyang wetland, located in Yangtze River delta in China (Figure 1). The wetland becomes an effective buffer and processor to the complex pollution of drinking water source which involves the removal of nutrients (Wang et al. 2012b), heavy metals (Wang et al. 2014), polycyclic aromatic hydrocarbons (PAHs) (Zheng et al. 2012b), and algae (Zheng et al. 2012a), as well as the promotion of bacteria community structure (Wang et al. 2011). Investigation of the long-term effects of accumulation of metals, especially heavy metals, has been conducted here (Wang et al. 2014). The results demonstrate that the combination of multilevel ponds and plant-bed/ditch systems can intercept and retain metals from source water. The achievements of this project in terms of ecological drinking water source practices were recognized by the presentation of several awards, including the Best Practices in Human Settlements Improvement in China in 2011 and the Dubai International Award for Best Practices to Improve the Living Environment in 2012.

More recently, the Changshuitang wetland in Haining became the site of the third CRCW (Figure 1). To further optimize the water treatment efficiency of this full-scale wetland (ca. 1.73 km^2^), a pilot wetland at a scale of approximately 1:100 (ca. 1.83 ha) was constructed in advance nearby and continued operating for over a year. The goals of the present study for this pilot wetland are: (1) To study the distribution of heavy metals and their accumulation effects on the wetland segments during initial period of pilot wetland, further to provide the guidance for the construction of full-scale wetland later; (2) To gain an enhanced understanding of the mechanism(s) on how the potential ecological health risks through purified raw water can be reduced even at low metal concentration.

## Results

### Metal contents and risk assessment in sediments/soil

#### Metal contents

The results of analysis of water (Additional file 1) and soil (Additional file 2) demonstrate that the pilot wetland exhibited considerable spatial heterogeneity between water (surface and bottom, as in Additional file 3) and soil (surface sediment and plant soils) samples after operating for over 1 year, particularly with respect to metals (Table 1). The spatial variations of water and soil quality indices suggests the presence of heterogeneity of water quality purification processes along the hydraulic flow pathways in the wetlands and variability in the enrichment effects for metals in the five functioning zones and structural units of the root-channel zone. For example, in the 84 sediment/soil samples, heavy metal contents were typically lower than those of major metals. The mean coefficient of variation (CV) for all metals in the pilot wetland was found to be 44.90%, with CVs decreasing in the following order: Ca > > Mg ≈ Na ≈ Zn > Pb > K ≈ Cd > Al > Cr ≈ Ni ≈ Cu > > Fe. The highest metal contents in the sediments of the wetlands exceeded the corresponding levels in source river sediments, suggesting that accumulation and enrichment did indeed occur within the wetlands, at least in some locations. The average contents of heavy metals in the composite source river sediment (Site A) are as follows (mg/kg): Cd: 0.09, Cr: 151.07, Cu: 32.34, Ni: 55.88, Zn: 167.52, Pb: 14.68. The maximum contents of all six heavy metals in the wetland soil/sediments (Sites B–G) exceeded the corresponding source levels at Site A without exception (average ratios: 2.37), with ratios (i.e., wetland/source) as follows: Cd: 3.08, Cr: 1.67, Cu: 1.22, Ni: 1.32, Zn: 2.55, Pb: 4.37. Despite sharing the same source, the metals exhibited considerable spatial variation throughout the wetland: we found that the contents were 0.17–4.62 (mean: 1.05, CV: 49.59%, median: 0.93) times the corresponding background levels in the surface soils of the Hangzhou–Jiaxing–Huzhou plain for heavy metals. Compared with the source river sediments, Cd, and Pb were enriched in the pilot wetland on the whole, although their contents in source river sediments were as low as the background levels. Moreover, contents of Cr, Ni, and Zn in source river sediments were already higher than the Hangzhou–Jiaxing–Huzhou plain background levels adopted here. Heavy metal contents in the sediments of the central channel (i.e., newly constructed channel and deposited sediments) were relatively high (approximately 0.93–2.50 times background levels, mean: 1.28), indicating some degree of enrichment. Sediment particulates settled with distance along the flow pathways in the central channel (typically over dozens of meters). In contrast, contents of heavy metals such as Cu, Ni, Zn, and Pb increased by 11.12%, 17.49%, 16.14%, and 34.03%, respectively, demonstrating that heavy metals were retained primarily in the latter part of the central channel. These results suggest that superficial sedimentation could act as a primary sink for heavy metals in the stream network of the plain.

**Table 1:** **Contents of metals in source river sediments, wetland sediments/soils as compared with regional background levels**

Heavy metal	Range mg/kg	Mean mg/kg	CV	Source mg/kg	Background mg/kg	Major metal	Range	Mean	CV	Source	Background
Cd	0.05–0.28	0.12	38.80%	0.09	0.15	K	1.16–8.50%	3.74%	38.83%	1.79%	2.03%
Cr	70.32–251.95	121.57	25.70%	151.07	77.55	Na	0.42–24.28%	8.10%	61.80%	7.37%	1.08%
Cu	12.33–39.33	22.07	21.33%	32.34	30.85	Ca	0.21–12.58%	3.03%	110.06%	4.34%	0.69%
Ni	21.74–73.70	38.93	23.52%	55.88	32.40	Mg	0.22–2.02%	0.70%	63.68%	0.95%	0.92%
Zn	1.96–427.93	112.95	61.64%	167.52	92.67	Al	0.97–16.84%	9.69%	33.25%	6.41%	7.57%
Pb	5.22–64.20	25.37	48.67%	14.68	30.35	Fe	2.45–4.93%	3.76%	11.53%	3.54%	3.23%

Note: *n* = 84; CV: coefficient of variation; Background levels are contents in the surface soils of the Hangzhou–Jiaxing–Huzhou plain (Wang et al. 2007).

#### Risk assessment of heavy metals

According to Environment Canada standards (MacDonald et al. 2000), two distinct thresholds can be used to assess pollution conditions in wetland sediments: the threshold effect level (TEL) and the probable effect level (PEL). Heavy metal contents lower than TEL are indicative of rare or minor pollution with little to no biological toxicity; thus, such pollution rarely induces negative ecological effects. In contrast, heavy metal contents that lie between TEL and PEL are indicative of moderate pollution that occasionally poses negative ecological risks. When heavy metal content exceeds PEL, serious pollution may occur and may be associated with considerable biotoxicity; in such instances, the negative ecological effects can be pronounced. All sediment samples collected in the pilot wetland exhibited Cd values that were below TEL, suggesting negligible Cd pollution in the wetland. However, the wetland was found to be slightly polluted by Cu, Pb, and Zn, with 98.81%, 88.10%, and 65.48% of sites between TEL and PEL. Cr and Ni contents exceeded PEL in 84.52% and 53.57% of sites. Thus, it can be assumed that heavy metals exhibit a generally low ecological risk in the pilot wetland, although Cr and Ni should be monitored closely. Further details are showed in Table 2.

**Table 2:** **Risk assessment of heavy metals in source river sediment, wetland sediments/soils, and regional background soils (mg/kg, dried weight)**

Heavy metals	TEL	PEL	Hangzhou–Jiaxing–Huzhou plain soil layer A	Source river	Within wetland, ***n*** = 84			Percentage		
					Max	Min	Mean	<TEL	TEL ~ PEL	> = PEL
Cd	0.596	3.53	0.152	0.09	0.28	0.05	0.12	100.0%	0.0%	0.0%
Cr	37.3	90	*77.6*	**151.07**	**251.95**	*70.32*	**121.57**	0.0%	15.5%	84.5%
Cu	35.7	197	30.8	32.34	*39.33*	12.33	22.07	98.8%	1.2%	0.0%
Ni	18	36	*32.4*	**55.88**	**73.70**	*21.74*	**38.93**	0.0%	46.4%	53.6%
Zn	123	315	92.7	*167.52*	**427.93**	21.96	112.95	65.5%	32.1%	2.4%
Pb	35	91.3	30.4	14.68	*64.20*	5.22	25.37	88.1%	11.9%	0.0%

Note: TEL: threshold effect level; PEL: probable effect level. The plain text, italics, and boldface regarding the contents represent rare, *occasional*, and **frequent** negative ecological risks, respectively. Hangzhou–Jiaxing–Huzhou plain soil layer A data are from (Wang et al. 2007).

### Distribution of metals in wetland sediments/soils

#### Spatial variations of metal contents

**Figure 2:**
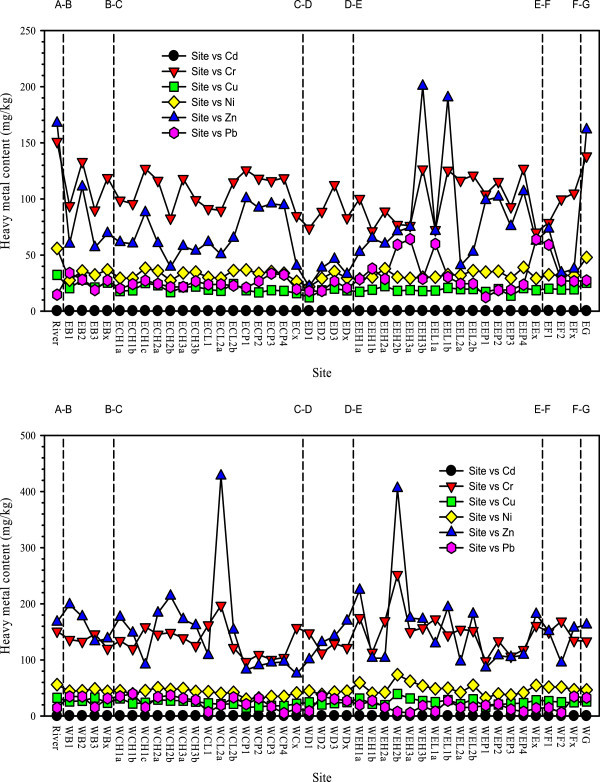
**Heavy metal contents in source river and locations of the east (top) and west (down) pilot wetland.** Refer to sampling location map in Figure 1. X-axis: River: source river (corresponding to “A” on top X-axis); the first letter “E”: east wetland, “W”: west wetland; the second letter (corresponding to letters on top X-axis) “B”: pretreatment zone, “C”: root-channel zone I, “D”: water lifting and falling zone, “E”: root-channel zone II, “F”: deep purification zone, “G”: wetland outlet; the third letter “H”: high ditch, “L”: low ditch, “P”: plant bed, “x”: the exit of functioning zone; the numbers after “B”, “D”, “F”: locations along hydraulic pathways; the numbers after “H”, “L”: ditch sequence; the numbers after “P”: plant bed sequence; the last letter “a”, “b”, “c”: locations along hydraulic pathways in ditches. Sites on plant beds are for collecting soil and the rest are for sediments.

Comparisons between the east and west blocks, the first and second parts, the five functioning zones along the hydraulic flow pathways, and the three finer scale structural units within both root-channel zones represent the basic characteristics of spatial variations within the wetland and are described here and in the following sections (Figure 2). The spatial distribution of metals in the east and west blocks was found to exhibit a complex pattern, which can be summarized as follows. (1) Cd exhibited obviously high contents in the east wetland, with average contents of 0.14 mg/kg (east) and 0.092 mg/kg (west). (2) Cr, Cu, Ni, and Zn contents were higher in the west wetland than the east, with average Cr contents of 103.51 mg/kg (east) and 141.00 mg/kg (west), average Cu content of 19.92 mg/kg (east) and 24.70 mg/kg (west), average Ni contents of 33.12 mg/kg (east) and 45.53 mg/kg (west), and average Zn contents of 75.67 mg/kg (east) and 152.77 mg/kg (west). (3) Pb contents varied little from east to west in the first part, but were higher in the east in the second part. These results demonstrate that, despite almost identical design and operation conditions in the east and west wetlands, nonparallel variations of metals were detected in wetland sediment, with no synergistic variation in the two successive parts of the pilot wetland.

#### Spatial variations of metal contamination factors

Contamination factor and enrichment factors (*EF*s) can be used to indicate metal pollution levels and are more suitable for characterizing retention processes than metal contents. The mean *EF*s of the six heavy metals studied were all considerably lower than 1.50, demonstrating that no obvious heavy metal accumulation (with respect to the low concentrations in the source river water) occurred on the whole during the initial operation period. However, the maximum *EF*s of Cd, Cr, Ni, Zn, and Pb exceeded 1.50, indicating some enrichment of these heavy metals in some parts of the wetland. Contamination factor (*C*) describes the contamination of a given toxic substance. The *C* value represented spatial variations in the pilot wetland. From data in Table 3, we can see contamination factors of most heavy metals in west block are higher than 1, which means the contents of heavy metals in pilot wetland were higher than the corresponding background level. Contamination factors of heavy metal Cd, Cu, Pb in two blocks were lower than 1 and most sites in the pilot had lower metal contents. Cr had 85.71% (average 1.32) and 100.00% (average 1.82) sample numbers that contamination factors were higher than 1 in east and west block, demonstrating the highest accumulation effects. Ni and Zn had similar variations whose contamination factors were higher in west block and more than half sites had contamination factors higher than 1. The maximum contamination factors for heavy metals were all above 1 except Cd in west and Cu in east. The studies proved that the primary operational pilot wetland had relatively accumulated the heavy metals, especially for Cr, Ni and Zn.

**Table 3:** **Statistics and proportion of heavy metal contamination factors (** ***C*** **) in wetland sediments/soils**

	Proportion (***C*** < 1)		Proportion (***C*** > 1)		Mean		Max	
	East	West	East	West	East	West	East	West
Cd	66.67%	100.00%	33.33%	0.00%	0.93	0.61	1.83	0.89
Cr	14.29%	0.00%	85.71%	100.00%	1.32	1.82	1.78	3.25
Cu	100.00%	88.10%	0.00%	11.90%	0.64	0.79	0.91	1.27
Ni	52.38%	4.76%	47.62%	95.24%	1.01	1.40	1.48	2.27
Zn	76.19%	11.90%	23.81%	88.10%	0.79	1.64	2.16	4.62
Pb	76.19%	69.05%	23.81%	30.95%	0.96	0.71	2.12	1.31

Note: Proportion means the number percentage of sample sites whose *C* met the demand in brackets accounting for the number of all sample sites.

Additional file 4 showed the heavy metal contamination factors in the pilot wetland. Generally, in east block, contamination factors for Cr, Cu, and Ni were high in pretreatment zone and factors for Cd and Pb were high in deep purification zone. In west block, contamination factors for Cd, Cu, Zn, and Pb were high in pretreatment zone, while factors for Cr, Ni were high in deep purification zone. The four root-channel parts had relatively high contamination factors, but lower than those at the outlets of wetland. Spatial variations of contamination factors in structural units (e.g., high/low water level ditch, plant bed) were investigated to analyze metal accumulation effects in root-channel zones (Table 4). The results reveal differences in contamination factors between the two blocks. In the east block, the contamination factors of Cd, Zn, and Pb in the ditches of the second part were higher than those in the first part, whereas the contamination factors of Cr, Cu, and Ni exhibited no distinct variance. The contamination factors of Cr, Cu, Ni, and Zn exhibited the opposite trend and those of Cd and Pb exhibited no clear variance. The contamination factors of Pb were lower in the plant bed of the second part; the contamination factors of Cd, Cr, Cu, Ni, and Zn exhibited the opposite trend. Moreover, contamination factors in low ditches were often lower than those in high ones.

**Figure 3:**
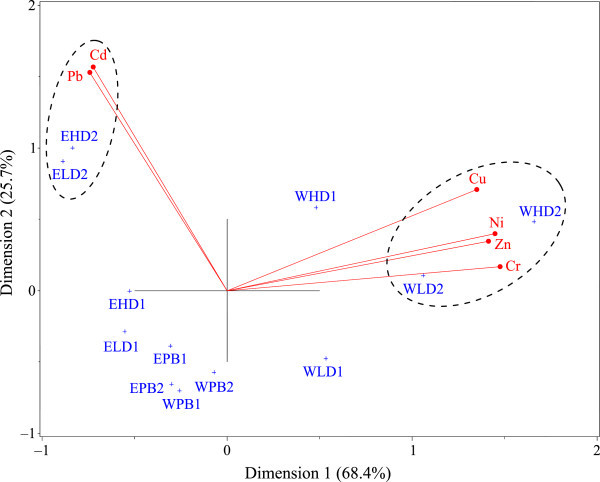
**Biplot of heavy metal contamination factors (** ***C*** **) in the plant-bed/ditch systems.** EHD, ELD, EPB: high ditch, low ditch, plant bed in east wetland; WHD, WLD, WPB: high ditch, low ditch, plant bed in west wetland.

**Figure 4:**
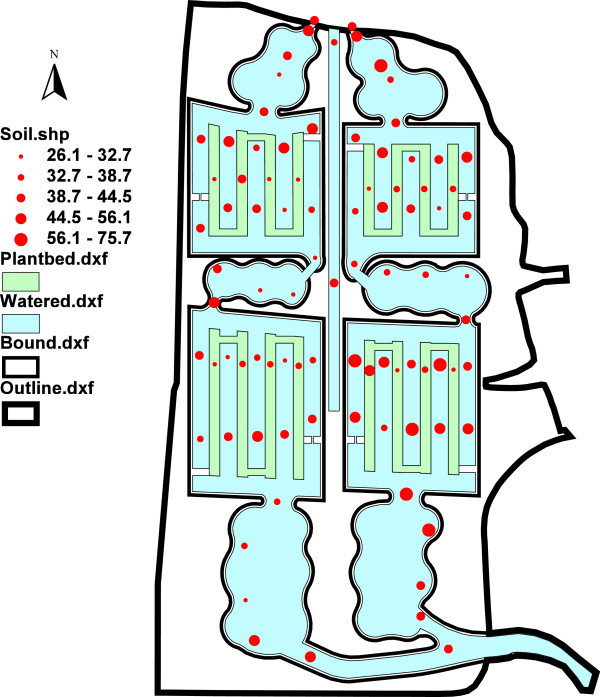
**Potential comprehensive ecological risk index (** ***RI*** **) of heavy metals in source river sediment, pond/ditch sediments and plant-bed soils of the pilot wetland.** Outline, bound line, watered line, plant bed line represent the outer boundary, inner boundary, watered area (light blue), and plant bed area (light green). The solid red circle illustrates the relative magnitudes of *RI*. The size range of circles is classified according to Natural Breaks method in ArcView GIS 3.2a.

The biplot of metal contamination factors (Figure 3) illustrates the multivariate correlations between variables (metal contamination factors) and observations (structural units). The explanatory percentage of first and second principal components accounted for 68.4% and 25.7%, respectively, explaining a total of 94.1% of the variance. This can be considered a very good fit. The results in Figure 4 demonstrate that no metals exhibited obvious enrichment in the plant beds of the first and second parts of either the east or west wetlands (i.e., all plant beds), or in the high/low ditches in the first part of the east wetland. The heavy metals Pb and Cd exhibited obvious accumulation in the high and low ditches in the second part of the east wetland; such accumulation was also detected for the heavy metals Cr, Cu, Ni, and Zn in the high and low ditches in the second part of the west wetland. In general, the plant-bed/ditch system in the Changshuitang pilot wetland accumulated heavy metals (Cd, Cr, Cu, Ni, Zn, and Pb) primarily in the second part of the wetland, with no apparent effects in the soil of the plant beds.

**Table 4:** **Contamination factors (** ***C*** **) of heavy metals in the structural units of root channel zones**

Block	Part	Unit	Cd	Cr	Cu	Ni	Zn	Pb
East	First	High Ditch	0.94 ± 0.19	**1.36 ± 0.2**	0.67 ± 0.1	**1.01 ± 0.13**	0.65 ± 0.16	0.78 ± 0.09
		Plant Bed	0.56 ± 0.02	**1.54 ± 0.05**	0.58 ± 0.03	**1.08 ± 0.04**	**1.03 ± 0.04**	0.93 ± 0.19
		Low Ditch	0.76 ± 0.07	**1.23 ± 0.17**	0.63 ± 0.11	0.94 ± 0.13	0.59 ± 0.12	0.74 ± 0.07
	Second	High Ditch	**1.18 ± 0.32**	**1.16 ± 0.27**	0.61 ± 0.06	0.96 ± 0.1	0.94 ± 0.6	**1.36 ± 0.54**
		Plant Bed	0.65 ± 0.11	**1.42 ± 0.19**	0.58 ± 0.1	**1.07 ± 0.13**	**1.03 ± 0.15**	0.61 ± 0.15
		Low Ditch	**1.24 ± 0.25**	**1.3 ± 0.35**	0.63 ± 0.03	0.99 ± 0.08	0.9 ± 0.66	**1.34 ± 0.65**
West	First	High Ditch	0.71 ± 0.15	**1.79 ± 0.17**	0.86 ± 0.1	**1.42 ± 0.11**	**1.77 ± 0.41**	**1.04 ± 0.26**
		Plant Bed	0.53 ± 0.06	**1.32 ± 0.07**	0.55 ± 0.04	**1.02 ± 0.07**	0.98 ± 0.07	0.61 ± 0.36
		Low Ditch	0.53 ± 0.15	**2.06 ± 0.4**	0.71 ± 0.02	**1.29 ± 0.04**	**2.06 ± 1.74**	0.6 ± 0.37
	Second	High Ditch	0.6 ± 0.14	**2.19 ± 0.59**	0.92 ± 0.23	**1.71 ± 0.39**	**2.13 ± 1.21**	0.51 ± 0.26
		Plant Bed	0.67 ± 0.07	**1.47 ± 0.2**	0.66 ± 0.09	**1.16 ± 0.12**	**1.1 ± 0.11**	0.49 ± 0.21
		Low Ditch	0.58 ± 0.15	**2.02 ± 0.14**	0.86 ± 0.11	**1.54 ± 0.16**	**1.69 ± 0.45**	0.52 ± 0.23

Note: mean ± standard deviation, boldface means data above 1.0.

#### Variations of risk index (RI) of heavy metals

By considering absolute contents and toxic response factors in tandem, the index *E* can indicate the potential ecological risk of an individual heavy metal, whereas the comprehensive potential ecological risk index (*RI*) represents the sum of the potential risk of all individual heavy metals. For individual metal elements in sediment/soil samples, most *E* values were considerably lower than 40, indicating low potential ecological risk (Håkanson 1980). Discernable spatial variations were also found in *RI* of heavy metals (Figure 4): *RI* for the six investigated heavy metals for the entire pilot wetland was calculated to be 26.13–75.74 (mean: 41.18) and was relatively high in the east (mean ± standard deviation, the same hereinafter) (44.39 ± 11.48) but low in the west (37.97 ± 6.69). In the east wetland, *RI* in the second part (47.10 ± 13.16) was higher than that in the first part (41.11 ± 8.25); conversely, in the west wetland, *RI* in the second part (37.81 ± 6.27) was approximately equal to that in the first part (38.17 ± 7.34).

Moreover, it is clear that *RI* exhibits similar patterns in both blocks, with significant decreases in the previous two functioning zones (i.e., the pretreatment and root-channel zones) owing to the effects of settlement in the pretreatment zone and to absorption and accumulation effects in root-channel zones. *RI* decreased gradually along the hydraulic flow pathways, with decreases of 29.93% and 35.70% from inlet to outlet in the first part in the east and west wetlands, respectively. After water pumping, *RI* increased sharply in both high and low ditches, exhibiting increases of 56.29% and 68.40% in the second part of the east and west wetlands, respectively. This demonstrates that both the pre-pond (at the front end of the wetland) and the post-pond (at the back end of the wetland) functioned effectively to retain metals under the push exerted by the water head and hydraulic flow. *RI* in the plant-bed/ditch systems of the root-channel zones exhibited spatial variation, decreasing in the following order: high ditch (45.35 ± 10.19), low ditch (41.09 ± 8.68), plant bed (33.67 ± 3.65). Small ditches within the plant-bed/ditch system in the root-channel zone appeared to cause enrichment by removing metals from source water. To summarize, we found the average *RI* to be higher than the mean of the whole wetland in all zones except the water lifting and falling zone, which had the lowest *RI*. Thus, heavy metals were largely retained along hydraulic flow pathways, helping to mitigate the ecological risk posed by the source water.

### Metal variations in wetland water

The concentrations of metals and total suspended solids (TSS) in wetland water samples were measured after sampling. According to Table 5, good removal rates were found for TSS in the pilot wetland, with removal rates of 47.80% and 37.58% in the east and west wetlands, respectively. This was likely the foundation for the removal of metals through deposition and adsorption. Mn in water samples was also measured and its concentrations were found to exceed the standard limited mean (0.10 mg/L) in both the inlet (source water) and the first part of the wetland (max: 0.17 mg/L); however, removal rates of Mn were high, with rates of 70.59% and 42.86% in the east and west wetlands, respectively. Cd, which has the strongest toxicity of the heavy metals examined, was not detected, because the water of the Jujinqiaogang River, which is adjacent to the Changshuitang pilot wetland, was micro-polluted. However, all other heavy metals investigated were detected. In the pilot wetland especially in the east block, concentrations of heavy metals declined sharply from sites B (E2,W2) to C (E3,W3) and from sites D (E4,W4) to E (E5,W5), implying that both root-channel zones in this block achieved good removal of heavy metals. Another obvious decline from sites A (E1,W1) to B (E2,W2) demonstrates that the pretreatment zone also exerted pronounced positive effects through mitigating the metal risk. This decrease corresponds well to changes in metal contents in sediments. Variations in TSS were also found to exhibit a good relationship with those of heavy metals.

**Table 5:** **Heavy metal contents in water column of pilot wetland along water flow pathways**

Site	TSS mg/L	Mn mg/L	Cd μg/L	Cr μg/L	Cu μg/L	Ni μg/L	Zn μg/L	Pb μg/L
E1	93.3	*0.17*	<1	2.27	4.31	1.49	*94.22*	3.00
E2	98.7	*0.14*	<1	1.66	3.51	0.93	25.10	2.29
E3	86.0	0.08	<1	1.00	3.38	0.74	18.23	2.62
E4	51.3	0.04	<1	1.20	2.59	0.52	21.29	1.91
E5	48.7	0.05	<1	0.64	3.48	0.21	15.42	1.72
E6	48.7	0.05	<1	1.72	4.04	0.32	39.99	2.22
*RR*	47.8%	70.6%	/	24.2%	6.3%	78.5%	57.6%	26.0%
W1	83.3	*0.14*	<1	1.11	2.90	0.99	41.44	2.01
W2	52.7	*0.15*	<1	2.18	5.86	1.14	39.16	4.07
W3	82.0	*0.12*	<1	2.19	3.98	1.13	19.47	2.28
W4	86.0	0.08	<1	2.13	5.26	0.63	18.94	2.04
W5	28.7	0.07	<1	2.39	4.67	0.48	21.37	2.67
W6	52.0	0.08	<1	2.37	2.87	0.33	16.73	1.86
*RR*	37.6%	42.9%	/	/	1.0%	66.7%	59.6%	7.5%
Limit	NA	0.1	≤1	≤10	≤10	20	≤50	≤10

Note: *RR*: removal rate; NA: not available; italic data are above the limits of basic indexes for grade I or supplementary indexes for centralized domestic drinking water sources according to Chinese environmental quality standards for surface water (GB 3838–2002).

Concentrations of heavy metals in all water samples were within the limits of basic indices for grade I according to Chinese environmental quality standards for surface water (GB 3838–2002), except in the case of Zn: Zn content at the eastern inlet (94.22 μg/L) exceeded the threshold (50 μg/L). Thus, ecological risk of heavy metals in the pilot wetland was rather small. The removal rates of Cu, Ni, Zn, and Pb were good, reaching 6.26%, 78.52%, 57.56%, and 26.00% in the east and 1.03%, 66.67%, 59.63%, and 7.46% in the west, respectively. The heavy metal Cr exhibited a good removal rate (24.43%) in the east but a lower rate in the west, although the rate in the west increased slightly with distance along the hydraulic flow pathways. However, these removal rates are less favorable than those of traditional wetlands (Bulc and Slak 2003; Tam et al. 2009), because nearly all heavy metal concentrations were within the standard prescribed limits. Nevertheless, it is clear that this novel type of constructed root-channel wetland has achieved good removal of trace metals in its initial period. Accordingly, such wetlands should be efficient in the mitigation of most heavy metal risks in source water.

### Contribution of aquatic plants to the uptake and accumulation of metals in wetland

Nine plant samples of six species of emerged aquatic plants were collected in the Changshuitang pilot wetland. Analysis of these plant samples indicated that aquatic plants had pronounced effects on the uptake and accumulation of metals (Table 6). The samples were collected without destroying plant rhizomes and roots, such that only the aboveground parts of the plant were collected. Compared with recent studies (Wu et al. 2013; Lesage et al. 2007; Bernard and Lauve 1995), the tissues of plants in the pilot wetland exhibited moderate heavy metal levels, with high contents of Cr, Cu, Ni, Zn, and Pb. We found reeds (*Phragmites australis*), the dominant wetland species, to have more pronounced effects than other plants on the uptake and accumulation of heavy metals, proving that plant uptake can contribute considerably to reducing heavy metals in the plant bed soils and partly explaining the relatively low heavy metal contents observed in the rhizospheric soil.

**Table 6:** **Heavy metals contents in above ground portion of plants in pilot wetland and other wetlands (mg/kg)**

Country	Wetland type	Plant species	Part	Cd	Cr	Cu	Ni	Zn	Pb	Reference
China	Root-channel wetland	*Schoenoplectus tabernaemontani*	AB	0.04	2.71	7.42	1.72	3.74	0.27	This study
		*Typha orientalis*	AB	0.03	1.70	2.81	0.70	0.91	0.58	This study
		*Typha orientalis*	RO	0.03	1.96	0.52	4.15	8.80	1.21	This study
		*Juncus effusus*	AB	0.17	2.58	6.21	1.21	13.77	1.30	This study
		*Acorus calamus*	AB	0.16	1.95	5.20	0.62	1.55	0.68	This study
		*Phragmites australis*	L	0.02	3.19	4.69	1.46	5.27	1.64	This study
		*Phragmites australis*	St	0.003	2.05	3.87	0.55	15.00	0.45	This study
		*Zizania caduciflora*	L	0.02	2.59	1.38	1.12	7.54	0.69	This study
		*Zizania caduciflora*	St	0.02	2.64	2.79	0.49	16.22	0.32	This study
China	Natural wetland	*Phragmites australis*	Sh	0.12-0.16	2.8-6.4	1.5-1.7	2.7-3.2	10.0-10.2	0.13-0.33	(Wu et al. 2013)
Belgium	Constructed wetland	*Phragmites australis*	St	0.031-0.083	0.9-1.3	2.2-4.4	0.20-0.52	11-39	0.34-0.43	(Lesage et al. 2007)
Belgium	Constructed wetland	*Phragmites australis*	L	0.028-0.071	0.72-0.95	0.91-3.3	0.29-0.45	20-29	0.53-1.1	(Lesage et al. 2007)
U.S.A.	Constructed wetland	*Phalaris arundinacea*	AB			1.9-7.1		10-28		(Bernard and Lauve 1995)

Note: AB: aboveground, L: leaves, St: stems, Sh: shoot, RO: reproduction organ.

## Discussion

### Metal accumulation effects in zones of CRCW

Constructed root channel wetland (CRCW) can remove many types of pollutants and plays an important role in water pollution control and the ecological rehabilitation of water environments (Wang et al. 2014). However, metals cannot be decomposed; rather, they must be changed morphologically to allow transportation and transformation of their special characteristics (González-Alcaraz et al. 2013; Yeh et al. 2009). When source water first enters the pilot wetland (i.e., in the pretreatment zone), the relatively wide water surface and slow hydraulic flow rates induce primarily the precipitation of TSS and organic matter. Owing to the special configuration of high/low ditches and plant-bed systems, metals can become further enriched in ditches and/or plant beds in root-channel zone I. In the present study, we found metal contents to be relatively low in these previous units, with little deposition in the water lifting and falling zone. The contamination factors of metals in root-channel zone II increased sharply with respect to those in previous zones. Metal deposition in the deep purification zone was much poorer than that in the root-channel zone, although the deep purification zone still promoted effective accumulation of metals owing to its slower hydraulic flow and wider water area. Previous studies have shown that, in constructed wetland, heavy metal contents in sediments typically decline gradually along hydraulic flow pathways (Obarska-Pempkowiak and Klimkowska 1999). Wetlands are believed to select their most favorable evolution direction in accordance with the surrounding environment. In the present study, metal content, contamination factors and *RI* were found to vary dramatically between five functioning zones, particularly between the two root-channel zones. Specifically, *RI* was found to first decline and then increase along the water flow pathways, reaching extremely high values in the two root-channel zones in the second part. This definitively proves that root-channel zones may become “hot sites” acting as sinks for heavy metals in this pilot wetland (Wang et al. 2014). Moreover, the results suggest that obvious deposition and accumulation of heavy metals occurred in CRCW, further highlighting the efficiency of this innovative technology for the mitigation of the ecological risk of metals.

Constructed root-channel wetlands are of particular importance because they can accumulate metals based on their specific characteristics. The retention of metals in the pilot wetland can be attributed primarily to sedimentation effects. Metals are likely to be combined with particulates in water after entering wetland areas, such that sediments are primary sinks for heavy metals (Zhao et al. 2012; Accornero et al. 2008; Sajwan et al. 2008). Also, the dominated aquatic plant reeds (*Phragmites australis*) played important role in sequestering metals (Weis and Weis 2004). In root channel zones, the rhizospheric microbial characteristics affected the metal accumulation (Wu et al. 2013). Phytoaccumulation occurs when heavy metals are not degraded in the plant, resulting in its accumulation within the plant tissues and the potential for plant uptake is highest in CWs due to the increased contact between water and the elaborate root systems of aquatic macrophytes (Malaviya and Singh 2012). In Tables 7 and 8, fluxes of heavy metals in sediment/soil, sedimentation and plant uptake masses of heavy metals in pilot wetland were calculated. Sedimentary mass of heavy metals accounted for 63.30% and soil part was 36.67%. However, percentage of metal mass by plant uptake was less than 0.1%. The pilot wetland can maintain about 86.18 kg total heavy metals since its operation. The fluxes for sediment ranged from 0.41-211.08 μg∙cm^-2^∙a^-1^, less than that in soils (0.73-543.94 μg∙cm^-2^∙a^-1^), both less than those in Shijiuyang Wetland (Wang et al. 2014).

**Table 7:** **Masses of heavy metals accumulated in pond/ditch sediments, plant bed soils and plants**

Structural unit	Type	Area/m ^2^	Cd/g	Cr/g	Cu/g	Ni/g	Zn/g	Pb/g	Sum/g	Percentage
Pond	Sediment	3615.43	20.19	9671.14	2412.70	3948.40	7237.27	3408.46	26698.15	30.98%
Ditch	Sediment	3554.16	20.68	10153.41	2370.52	3995.47	8133.73	3182.92	27856.72	32.32%
Plant bed	Soil	1740.14	17.26	12810.70	2917.46	5144.17	7878.62	2832.86	31601.07	36.67%
First part	Plant	735.40	0.07	1.65	3.20	0.97	5.58	0.72	12.19	0.01%
Second part	Plant	1004.74	0.03	2.46	3.89	1.50	7.70	0.68	16.26	0.02%
Total			58.22	32639.36	7707.76	13090.50	23262.91	9425.64	86184.39	100.00%

**Table 8:** **Accumulation fluxes of heavy metals in pond/ditch sediments and plant bed soil (μg∙cm** ^**-2**^ **∙a** ^**-1**^ **)**

Structural unit	Type	Operational days	Cd	Cr	Cu	Ni	Zn	Pb
Pond	Sediment	494	0.41	197.64	49.31	80.69	147.90	69.66
Ditch	Sediment	494	0.43	211.08	49.28	83.06	169.09	66.17
Plant bed	Soil	494	0.73	543.94	123.88	218.42	334.53	120.28

The east and west blocks of the wetland described here were designed with almost identical theoretical operating conditions, with similar characteristics in terms of construction, operation, and local plant conditions, yet they resulted in varied soil/sedimentary metal contents. However, despite the almost symmetrical and parallel design of the east and west blocks, the results presented here indicate non-parallel enrichments effects between the two blocks. This can be attributed to slight differences in local conditions, development, succession, human activities, and hydraulic factors (Stead-Dexter and Ward 2004; Wu et al. 2013). However, a more full characterization of these differences and their effects will require long-term comparative study, which will help to improve understanding of the system and allow optimization of engineering design and operation and management practices, thus allowing high purification efficiencies to be achieved consistently.

### Metals accumulation effects during operating periods of CRCW

The Changshuitang wetland is located within a dense stream network. Accordingly, it is subjected to extremely complex water sources, various pollutant types, and serious nonpoint and diffuse but small decentralized point source pollution (Yin et al. 2010). Pollutants such as heavy metals enter the water body of stream networks through overland runoff in cities. Previous studies have shown that the first full-scale CRCW with a similar structural design to that of the present study, the Shijiuyang wetland, demonstrated high enrichment factors for Cd, Cr, Cu, Ni, Zn, and Pb; these values remained above the threshold value of 1.5 after 4 years of operation (Wang et al. 2014). Both studies showed that the crisscrossed plant-bed/ditch systems in the root-channel zones were “hot sites” for the sedimentation, interception, enrichment, and even uptake of heavy metals in CRCW. Moreover, the risks of heavy metals were greatly mitigated through the complex interactions of the plant-bed/ditch systems. The removal rates of heavy metals in the water phase correspond well to the characteristics of the sediment and soil matrix (particularly particle size composition and organic matter content), and the interactions among plant-bed soil matrixes and the bilateral ditch water are capable of removing most species of metals, in conjunction with the deposition and filtration of coarse/finer particles.

Although the Changshuitang pilot wetland has been operational for only 1 year and performed poorly compared with the Shijiuyang wetland in terms of plant diversity and metal removal rate, it achieved better water quality using a more reasonable design for similar environmental and soil matrix conditions. In particular, its unique design helped enhance removal rates for metals and heavy metals. However, no extremely obvious decline of *RI* was observed in the pilot wetland, particularly compared with that between the wetland inlet (Site A, source water sediment) and the wetland outlet (Site G, exit of deep purification zone). This may be due to instability of wetland structure and function, low biodiversity, and the unstable effects of water purification (Saeedreza et al. 2012). The effects of metal accumulation in the pilot wetland’s primary operation (one-year period) were comparatively lower than those in the relatively developed Shijiuyang wetland (four-year period). In addition, it may be important that the hydraulic flow pathways (direct length ca. 200–300 m) and hydraulic retention time (ca. 2.3 d) are both relatively small under a hydraulic flow rate of 0.27 m/d. This may be insufficient for thorough interaction between water and sediments/soil (Bilal et al. 2009). Moreover, the very low concentrations of metals (except Zn) in the source river water do not provide a considerable pollution load to the wetland, where the optimum treatment efficiency of such metals may be achieved under conditions in which an allochthonous source input is present. At present, *RI* in the pilot wetland is far below the threshold value of 150, indicating little environmental damage. Nevertheless, metal contents at the outlet of the pilot wetland remain relatively high owing to the effects of accumulation; this can be attributed to the internal interweaved “capillary” filtration functions, which can be visualized by considering the whole pilot wetland as the glomerulus of a kidney. Decreases in flow velocity at the restricted exit of the wetland will promote metal deposition accompanied by particulate settling (Bilal et al. 2009; Brix and Arias 2005). Nonetheless, more long-term monitoring data will be required to assess the actual removal of metals and the transformation of metal forms. Moreover, further emphasis should be placed on the effects of long-term operation, accumulation effects, the use of aquatic plants, and the effects of novel CRCW techniques on the removal of metals (especially heavy metals) in future.

## Conclusions

The metals exhibited considerable spatial variation within the pilot wetland. According to the generally adopted sediment quality assessment values for freshwater ecosystems, the pilot wetland showed rare or occasional adverse effects by heavy metals. Most sample sites in the wetland had contamination factors above one and the maximum contamination factors for heavy metals were all above one except Cd in west wetland and Cu in east wetland. As contamination factors showed, the plant-bed/ditch system primarily in the second part of the Changshuitang pilot wetland accumulated heavy metals (Cd, Cr, Cu, Ni, Zn, and Pb). The comprehensive potential ecological risk index (*RI*) decreased gradually along the hydraulic flow pathways in the first part and increased in the second part indicating that both the pre-pond and the post-pond functioned effectively to retain metals. Small ditches within the plant-bed/ditch systems in the root-channel zone demonstrated the ability to remove metals from source water. *RI* exhibited spatial variation, decreasing in the following order: high ditch, low ditch, plant bed. Heavy metals in the source water became considerably accumulated and enriched within the plant-bed/ditch systems in the root-channel zones. The wetland effectively eliminated Cu, Ni, Zn, and Pb, with high removal rates. Sedimentary mass of heavy metals accounted for 63.30% and soil part was 36.67% in this wetland. However, percentage of metal mass by plant uptake was less than 0.1%. The fluxes for sediment ranged from 0.41-211.08 μg∙cm^-2^∙a^-1^, less than that in soils (0.73-543.94 μg∙cm^-2^∙a^-1^). The pilot wetland can maintain about 86.18 kg total heavy metals since its operation. This pilot case study proves that a purification mechanism formed by a combination of multilevel ponds and plant-bed/ditch systems can retain heavy metals from the micro-polluted source water, even during its first year of operation, thus reducing the potential ecological risk of the purified raw water. The pre-pond, plant-bed/ditch, and post-pond complexes can provide a new and effective method for the removal of heavy metals in drinking water sources.

## Materials and methods

### Background and study area

The water source in the river networks of the Yangtze River delta of China has become increasingly polluted in response to rapid economic development and urbanization within the watershed. Jiaxing belongs to the delta with a population of 4.5 million, where heavy metal is one of the potentially important environmental issues. Many industrialized processes including dying, plating, and tanning give rise to the contamination by heavy metals, such as cadmium (Cd), mercury (Hg) and lead (Pb) in soil, water and air (Huang et al. 2013). Busy land and water transportation in the Great Canal are also an important input source for the stream network. Urban runoff caused by rainfall on city dusts is one of the primary pathways of heavy metal into the stream network, and the narrow land/water boundaries, absence of buffer zones, and ineffective street cleaning methods contribute to the heavy metal inputs to the stream network (Zhao et al. 2009). Jiaxing has no mountain reservoir for water source and has to take drinking water from river network. Although considerable efforts have been made to control the pollution in the city’s stream network, some of the drinking water in the network remains polluted owing to the complex conditions in the study area, thus threatening water safety in the region (Yin et al. 2010).

On April 1, 2007, Jiaxing began to construct the Shijiuyang large-scale ecological water quality treatment wetland (Figure 1), which was intended to purify and restore the healthy drinking source water. This treatment system was based on three patented technologies. The core technology focused on constructing root-channels artificially in constructed wetland and was developed by the Research Center for Eco-Environmental Sciences (RCEES), Chinese Academy of Sciences (CAS) (Wang et al. 2012a). The trial operation of the wetland was initiated on July 2, 2008. This full-scale wetland incorporated root-channel renewability to prevent clogging. To date, the wetland has been operating continuously for nearly 6 years realizing the goal of improving source water quality by one level according to Chinese environmental quality standards for surface water (GB 3838–2002). Furthermore, the wetland has become a city park serving multiple ecological functions. This innovative technique put into practice by Jiaxing both guarantees drinking water safety for the local population and promotes continuous improvement of the ecological and living environments. Moreover, it provides a meaningful example for other cities that may be facing similar problems relating to micro-pollution of their drinking water.

Thanks to the success of the Shijiuyang wetland, the Changshuitang wetland was constructed in Haining, becoming the third CRCW in China (Figure 1). The Changshuitang wetland benefited from many improvements over the Shijiuyang wetland. For example, it benefited from the design and application of many optimization measures, including increased diversity of pioneer plants, application of two-way parallel water flow pathways, strengthening of hydraulic control measures, and adoption of a step-feed process of water supply to enhance ammonia and organic matter removal. These measures were adopted primarily to further mitigate the complex pollution arising from industrial, domestic, and agricultural sources in the drinking water sources represented by the Changshanhe and Changshuitang rivers, which are treated by the Third Water Plant in Haining. The Changshuitang wetland is located in the northern part of Haining, which lies to the east of the Third Water Plant. The wetland covers an area of about 1.73 km^2^ and aims to purify about 0.3 million m^3^ of drinking water per day. The wetland itself was designed to use the natural topography of the original course of the river, based on the concept of ecological treatment, and was constructed according to the patent technologies of RCEES, as described above. Today, the wetland is still under construction.

To further optimize, promote, and verify the water treatment efficiency of the full-scale wetland (ca. 1.73 km^2^), a pilot wetland at a scale of approximately 1:100 (ca. 1.83 ha) was built beforehand nearby from March to June 2011. Its geographical coordinates span 30°34′5.36″–30°34′11.82″ N and 120°42′23.32″–120°42′27.79″ E. It was designed as two parallel wetland blocks divided by a central water supply channel that serves as a partial water source (i.e., accounting for 20 - 50% of the total water supply amount) to allow step feeding of the wetland through the pump station and through horizontal water pipes in the first and second parts, respectively (Figure 1). This configuration results in east and west wetlands for separate purification of the micro-polluted drinking water source. Each block (wetland) is composed of five functioning zones, which are further divided into two parts by a pump station. The first part is located before the pump station and contains pretreatment zone and root-channel zone I, whereas the second part lies after the pump station and contains the water lifting and falling zone, root-channel zone II, and the deep purification (polishing) zone. Each root-channel zone consists of finer scale structural units including a high water level ditch (high ditch), low water level ditch (low ditch), and plant bed. The pilot wetland can deal with 1880 m^3^ water per day and typically improves water quality by one level. To help understand the water depth distribution in functioning zones and water flow movement through the wetland, the section and elevation planning of the pilot wetland was presented in Additional file 5.

### Sample collection and preparation

In total, 71 sediment samples from ponds/ditches and 16 soil samples from plant beds were collected on December 7–8, 2012 (Figure 1). We assigned sample sites in representative areas of the wetland to obtain information regarding key processes. As the area of pilot wetland was small, boats could not be used to collect samples. Then sample sites in water lifting and falling zone as well as deep purification zone were not in the middle part but we try our best to collect the most representative samples. The sampling site numbers were assigned as follows. Single “C” before site names indicates sites in the central channel, with C1 for the inlet and C2 in sites beside the water pump stations controlling the step-feed process. Similarly, the use of prefix “W” and “E” before site names indicate sites in the west and east blocks, respectively. Two equivalent sedimentary samples from the inlets of the east and west sides were mixed as one; this sample is labeled “Source” (or “A”). According to the hydraulic flow pathways, the first part lies before the pump station and contains pretreatment zone (B) and root-channel zone I (C), whereas the second part includes the water lifting and falling zone (D), root-channel zone II (E), and the deep purification zone (F). The outlets of the wetlands are indicated by “Outlet” (or “G”). Within each zone, we used numbers to label different sites along the water flow pathways. In each root-channel zone, the ditches lead the water deep into the wetland and let the water penetrate through the root-channels under the plant beds. “H” and “L” refer to ditches with high and low water levels, respectively, and “P” indicates sites of soil samples from plant beds.

About 400–500 g wet weight of mixed surface sediments (0–10 cm) were collected using an Ekman bottom sampler (Hydro-Bios, Kiel, Germany). To reduce the heterogeneity of sediments and avoid the effects of water and plant, three equal samples were collected and mixed as one. Soil samples from plant beds weighing 300–400 g were collected using a soil auger (XDB-TR7, sampling depth: 1.5 m, drill diameter: 5 cm; Beijing New Landmark Soil Equipment Co., Ltd.) at depths of 0–30 cm. Immediately after collection, soil temperature, pH, and redox potential (*E*_h_) were measured using an IQ150 probe (HACH, USA); then, samples were preserved in bags on ice for transportation. Samples for ammonium-N, nitrate-N, and nitrite-N assays were stored under -4 °C. The remaining samples were frozen, air dried, and sieved through a 100-mesh sieve to remove coarse particles. The sediment/soil samples were then stored in plastic bags for further analysis.

Twelve water and nine plant samples were collected on August 3, 2013 for further investigation of metal contents and risk levels in the water column and plants of the wetland in full flourish. Water samples are indicated by “W” (west wetland) or “E” (east wetland) and numbers, while plant samples are expressed by “P” and numbers. Samples W1–6 and E1–6 represent key sites at the intersections between two functioning zones, i.e., the inlet of the wetland (where the source water feeding the wetland enters the system), outlet of the pretreatment zone, outlet of root-channel zone I, outlet of the water lifting and falling zone, outlet of root-channel zone II, and outlet of the deep purification zone (i.e., the exit of the whole wetland). Water samples for the analysis of metal contents were stored after filtering through 0.45-μm filter membranes before adding HNO_3_. General water samples were stored at cold temperatures after their temperature, pH, dissolved oxygen (DO), and redox potential (*E*_h_) had been analyzed. All types of emerged aquatic plants present were sampled to provide a representative indication of plant conditions in the wetland; such plant samples were stored in plastic bags after determination of their stem diameter, height, and density from random samples.

### Analysis and quality control

For metal extraction, 0.1-g samples of dried soil were digested in 6 mL of an HNO_3_ and HCl mixture (1:3) and 2 mL HF in a CEM microwave (MARS Xpress; CEM, USA) according to the program recommended by the United States Environmental Protection Agency (EPA) (Bettinelli et al. 2000). These transparent solutions were then filtered through 0.45-μm filter membranes and diluted to 50 mL with distilled water. The concentrations of metals Cd, Cr, Cu, Ni, Pb, and Zn in the filtrate were determined by ICP–MS (7500a; Agilent, USA), whereas the concentrations of metals K, Na, Ca, Mg, Al, and Fe were measured by ICP–OES (OPTIMA 2000DV; Perkin Elmer, USA).

Determination of metal content of water samples was achieved by ICP - OES (OPTIMA 2000DV; Perkin Elmer) for K, Na, Ca, Mg, and Fe and by ICP - MS (7500 a; Agilent) for Al, Cd, Cr, Cu, Ni, Zn, and Pb. Total suspended solids (TSS) were measured by mass according to standard methods. Plant samples were digested by HNO_3_ - H_2_O_2_ in a CEM after drying and sieving; further details are provided in the existing literature (Niemelä et al. 2004). The subsequent metal determination methods for plant samples were the same as those for soil/sediment samples.

The metals can be classified into groups/types according to several classification systems in the context of study objectives. To ensure clarity, we refer to the metals K, Na, Ca, Mg, Al, and Fe as major metals owing to their abundance in the study area (their contents are given in %) and refer to the metals Cd, Cr, Cu, Ni, Zn, and Pb (i.e., those measured in mg/kg) as heavy metals, following the most commonly used terminology. Major metals were not the wide discussion in this study with only necessary description.

### Enrichment factor

The enrichment factor (*EF*) is typically used to evaluate sources of metals and requires the use of a standard element that fulfills several criteria (Abrahim and Parker 2008). We adopted Fe as the standard element (Cobelo-García and Prego 2003) and calculated *EF* as follows.
1

Here, (Me*/*Fe)_sample_ and (Me*/*Fe)_background_ refer to the ratios of a target metal to that of Fe in a soil/sediment sample and in the background, respectively. We adopted metal contents obtained for the surface soil in the Hangzhou–Jiaxing–Huzhou plain (soil layer A) as the background content (Wang et al. 2007). Higher values of *EF* indicate more extensive accumulation of metals. *EF* < 1.5 signifies that metal contents are natural levels; thus, *EF* > 1.5 suggests that metals resulting from human activities are a key component (Håkanson 1980).

### Assessment of potential ecological risk index of heavy metals

The potential ecological risk index (*RI*) of heavy metals can be used to assess the ecological risk they pose. Such a risk is typically assessed using an index that reflects the content of heavy metals, the number of heavy metal pollutant sources, the toxicity level, and any ecological/environmental effects, as follows (Håkanson 1980).
2

Here,  and  represent the content of heavy metal *i* and its background content in Hangzhou–Jiaxing–Huzhou plain (soil layer A), respectively. Thus,  (i.e., ) represents the contamination factor of heavy metal *i*.  indicates the toxic response factor for a given heavy metal, where  is 30, 2, 5, 5, 1, and 5 for Cd, Cr, Cu, Ni, Zn, and Pb, respectively (Håkanson 1980),  represents the potential ecological risk index of an individual heavy metal, and *RI* is the sum of the potential risk of all individual heavy metals.

### Fluxes and masses of heavy metals

Flux of heavy metals in sediment/soil can be calculated with indices such as content of metal, moisture content and dry bulk density (Wang et al. 2014). It represents the rate of sedimentary effects on heavy metals.
3

Here, *F*_*i*_ means fluxes of heavy metal *i* in sediment/soil (mg∙m^-2^∙d^-1^), *C*_*i*_ means contents of metal *i* (mg∙kg^-1^); *W* indicates moisture content; *ρ* indicates average dry bulk density (kg∙m^-3^); ∆*H* indicates thickness of sediment/soil (m); *t* represents operational days (d).

Mass of heavy metals accumulated or absorbed by sediment/soil and plants is calculated by formula 4 and 5.
4

Here, *M*_*i*_ indicates accumulation mass of metal *i* (mg); *S*_*j*_ means area of zone *j* (m^2^).
5

Here,  indicates uptake mass of heavy metal *i* by plants (mg);  means content of metal *i* in plants (mg∙kg^-1^); *B*_*j*_ indicates biomass of plants (kg∙m^-2^);  means area of aquatic plants (m^2^); *n* represents times of reaping.

### Statistical analysis

Data analysis was performed using SAS for Windows 9.2 (SAS Institute, Inc., Cary, NC, USA) (Friendly 1991; Friendly 2000). Unless otherwise stated, *α* = 0.05 and *α* = 0.01 were adopted as the statistically significant and extremely significant levels. A biplot was drawn using the IML module in the SAS system to visualize the multivariate relationships between observations and variables. In this plot, the lengths of environmental vectors represent the ability to distinguish different variables and the cosine of angles between two vectors expresses the degree of correlation. All variables in this biplot were standardized to ensure consistency in units and dimensions.

## Electronic supplementary material

Additional file 1: **Statistics of basic water quality indices in the pilot wetland.** Statistics as range, mean, standard deviation, and coefficient of variation were performed on water depth and general water quality indices determined in situ. The results are arranged by block, part, and layer. (XLS 35 KB)

Additional file 2: **Statistics of basic sedimentary/soil quality indices in the pilot wetland.** Statistics as range, mean, standard deviation, and coefficient of variation were performed on general soil quality indices determined in situ and indoors. The results are arranged by block and part. (XLS 32 KB)

Additional file 3: **Distribution of dissolved oxygen saturation in water column of the pilot wetland.** (a) Surface layer, View 1; (b) bottom layer, View 2. The surface (grid) interpolation is performed according to IDW method based on nearest neighbors in ArcView GIS 3.2a. The graduated color of dissolved oxygen saturation is classified by equal intervals and illustrated with color ramps of full spectrum. (PDF 88 KB)

Additional file 4: **Heavy metal contamination factors in source river and locations of the east (top) and west (down) pilot wetland.** Refer to sampling location map in Figure 1. X-axis: River: source river (corresponding to “A” on top X-axis); the first letter “E”: east wetland, “W”: west wetland; the second letter (corresponding to letters on top X-axis) “B”: pretreatment zone, “C”: root-channel zone I, “D”: water lifting and falling zone, “E”: root-channel zone II, “F”: deep purification zone, “G”: wetland outlet; the third letter “H”: high ditch, “L”: low ditch, “P”: plant bed, “x”: the exit of functioning zone; the numbers after “B”, “D”, “F”: locations along hydraulic pathways; the numbers after “H”, “L”: ditch sequence; the numbers after “P”: plant bed sequence; the last letter “a”, “b”, “c”: locations along hydraulic pathways in ditches. Sites on plant beds are for collecting soil and the rest are for sediments. (PDF 78 KB)

Additional file 5: **Sectional schematic diagram and elevation planning of the pilot wetland.** The normal water level of source river is 0.990 m according to Huanghai Vertical Datum 1985. After pretreatment zone and root-channel zone I, the water level decreases 0.05 m and 0.20 m respectively. By virtue of pump station lifting, the partly treated water is lifted 1.05 m, and two cascades make the water level drop 0.30 m altogether. After root-channel zone II, the water level decreases 0.30 m. Then water level drops another 0.20 m through the deep purification zone and falls back to the normal water level at the outlet of the wetland. The pilot wetland is composed of ponds, ditches, and plant beds with various sizes and depth. (PDF 2 MB)

## Abbreviations

CRCW: Constructed root-channel wetland
TEL: Threshold effect level
PEL: Probable effect level
*EF*: Enrichment factor
*RI*: Comprehensive potential ecological risk index
TSS: Total suspended solids
CV: Coefficient of variation.
