# Supplementary figures and images for: Retention and mitigation of metals in sediment, soil, water, and plant of a newly constructed root-channel wetland (China) from slightly polluted source water
Source: Springerplus. 2014 Jun 28;3:326. doi: 10.1186/2193-1801-3-326 (PMC4094763; doi:10.1186/2193-1801-3-326)

**Outline.shp**  
**Surface from Do\_s.shp**

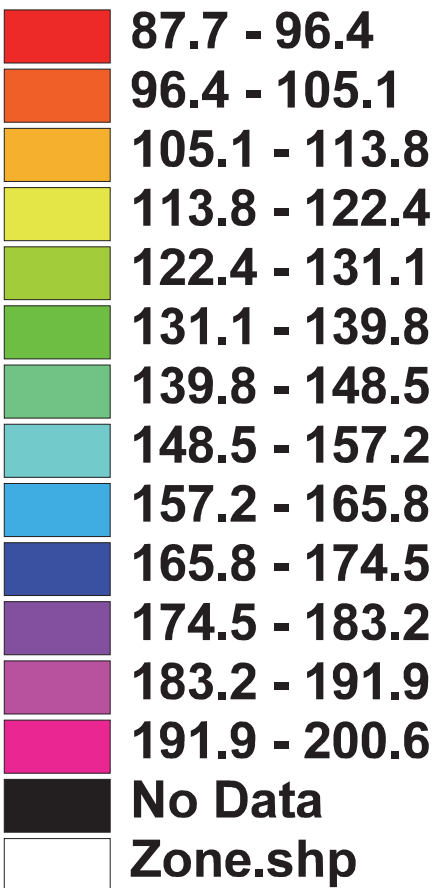

**View1**

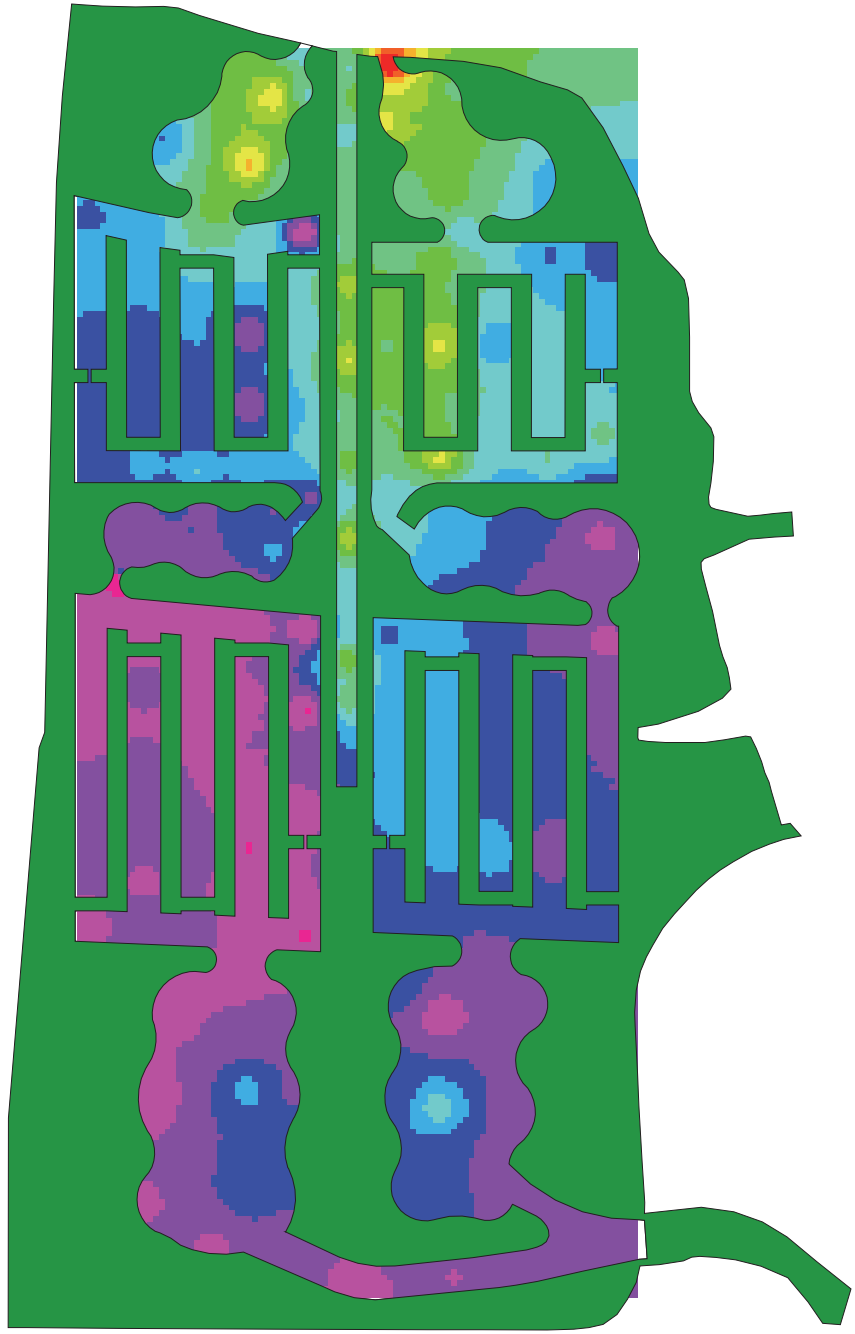

**Outline.shp**  
**Surface from Do\_b.shp**

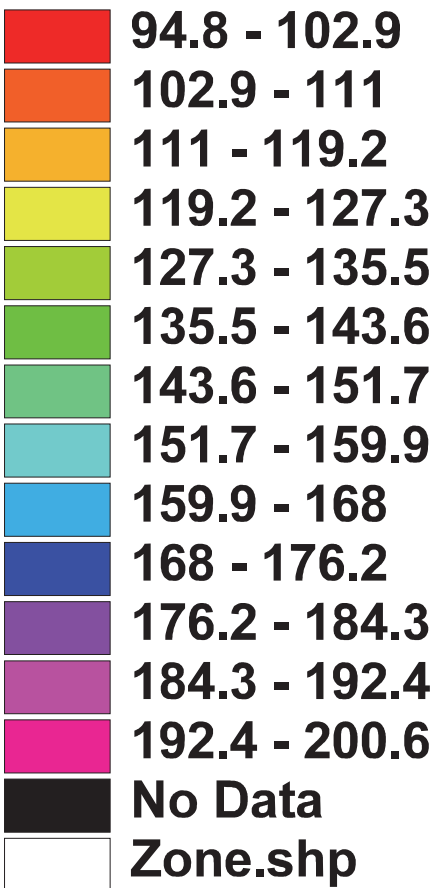

**View2**

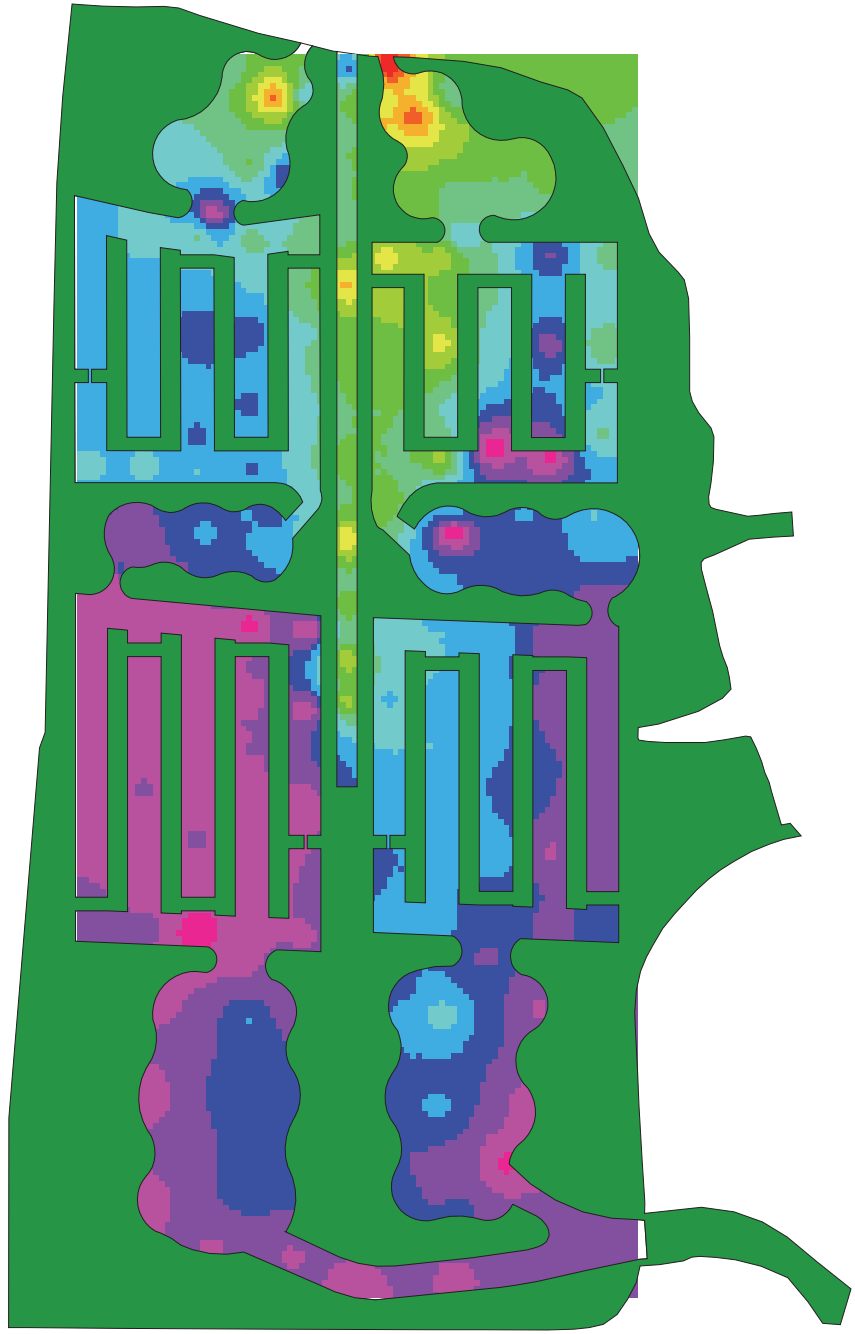

Supplement: Supplementary file 3 — Additional file 3: Distribution of dissolved oxygen saturation in water column of the pilot wetland. (a) Surface layer, View 1; (b) bottom layer, View 2. The surface (grid) interpolation is performed according to IDW method based on nearest neighbors in ArcView GIS 3.2a. The graduated color of dissolved oxygen saturation is classified by equal intervals and illustrated with color ramps of full spectrum. (PDF 88 KB) [file 40064_2014_1040_MOESM3_ESM.pdf]

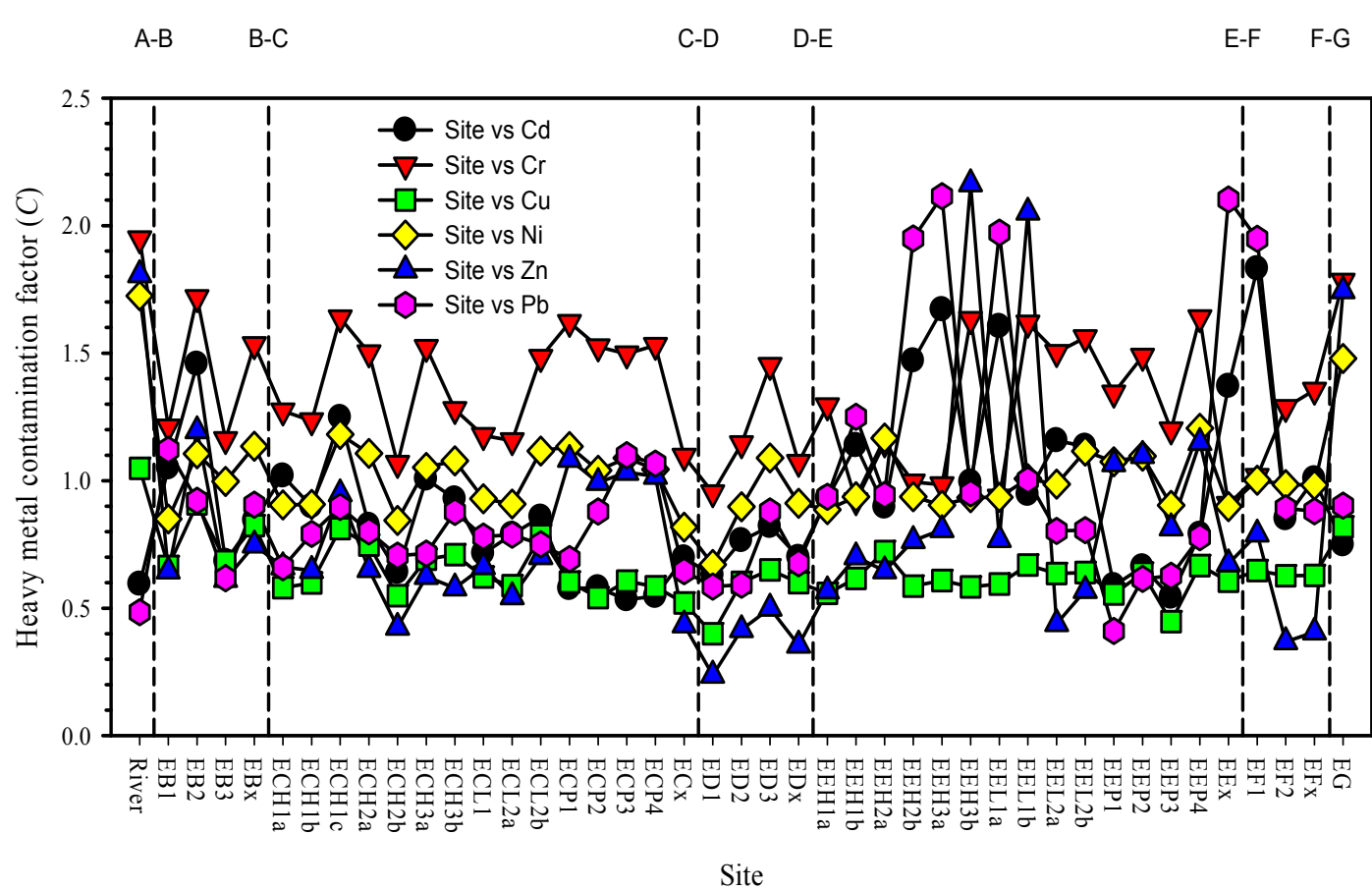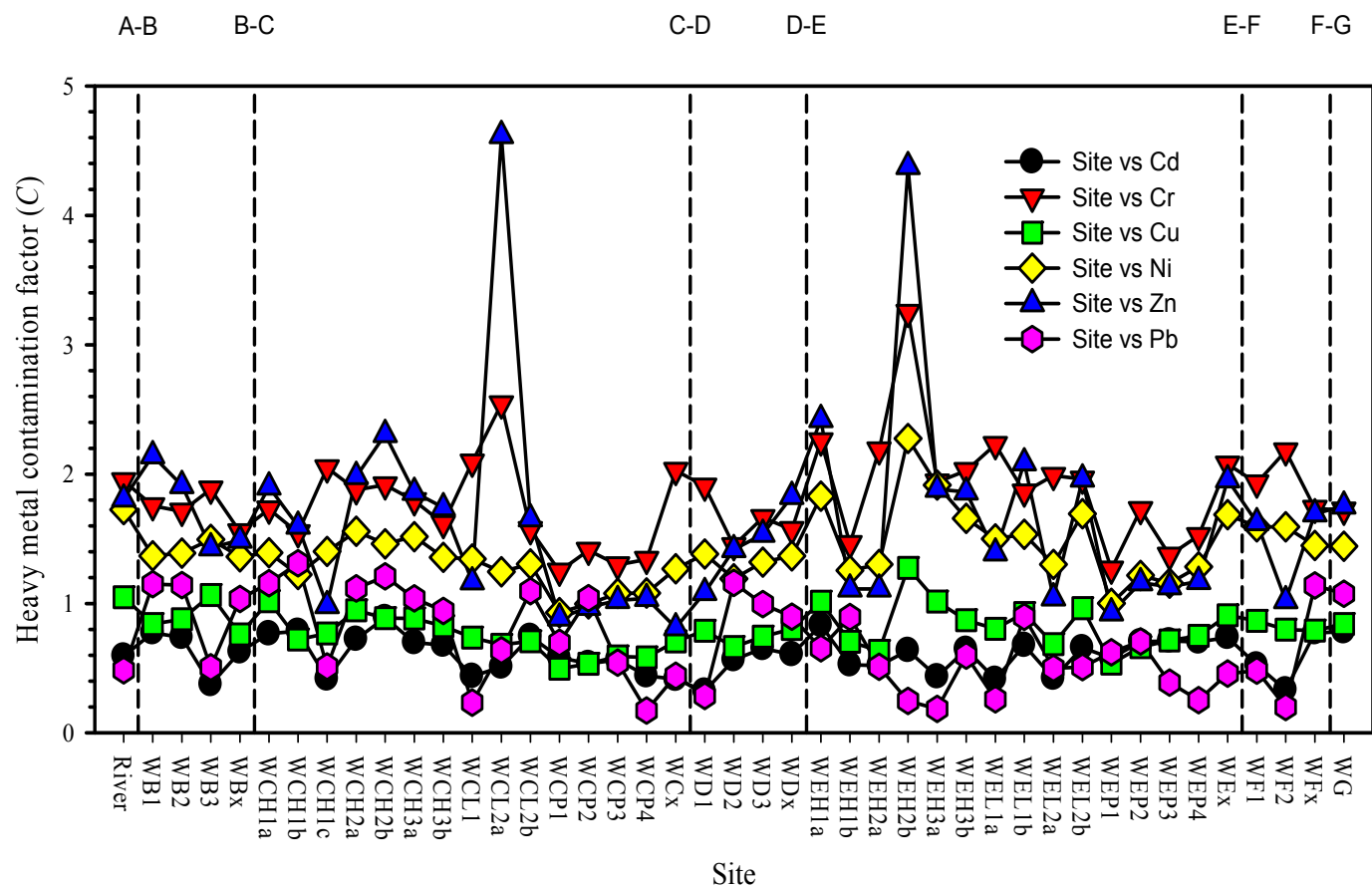

Supplement: Supplementary file 4 — Additional file 4: Heavy metal contamination factors in source river and locations of the east (top) and west (down) pilot wetland. Refer to sampling location map in Figure 1. X-axis: River: source river (corresponding to “A” on top X-axis); the first letter “E”: east wetland, “W”: west wetland; the second letter (corresponding to letters on top X-axis) “B”: pretreatment zone, “C”: root-channel zone I, “D”: water lifting and falling zone, “E”: root-channel zone II, “F”: deep purification zone, “G”: wetland outlet; the third letter “H”: high ditch, “L”: low ditch, “P”: plant bed, “x”: the exit of functioning zone; the numbers after “B”, “D”, “F”: locations along hydraulic pathways; the numbers after “H”, “L”: ditch sequence; the numbers after “P”: plant bed sequence; the last letter “a”, “b”, “c”: locations along hydraulic pathways in ditches. Sites on plant beds are for collecting soil and the rest are for sediments. (PDF 78 KB) [file 40064_2014_1040_MOESM4_ESM.pdf]
