# Supplementary material for: Retention and mitigation of metals in sediment, soil, water, and plant of a newly constructed root-channel wetland (China) from slightly polluted source water
Source: Springerplus. 2014 Jun 28;3:326. doi: 10.1186/2193-1801-3-326 (PMC4094763; doi:10.1186/2193-1801-3-326)
Supplement: Supplementary file 5 — Additional file 5: Sectional schematic diagram and elevation planning of the pilot wetland. The normal water level of source river is 0.990 m according to Huanghai Vertical Datum 1985. After pretreatment zone and root-channel zone I, the water level decreases 0.05 m and 0.20 m respectively. By virtue of pump station lifting, the partly treated water is lifted 1.05 m, and two cascades make the water level drop 0.30 m altogether. After root-channel zone II, the water level decreases 0.30 m. Then water level drops another 0.20 m through the deep purification zone and falls back to the normal water level at the outlet of the wetland. The pilot wetland is composed of ponds, ditches, and plant beds with various sizes and depth. (PDF 2 MB) [file 40064_2014_1040_MOESM5_ESM.pdf]

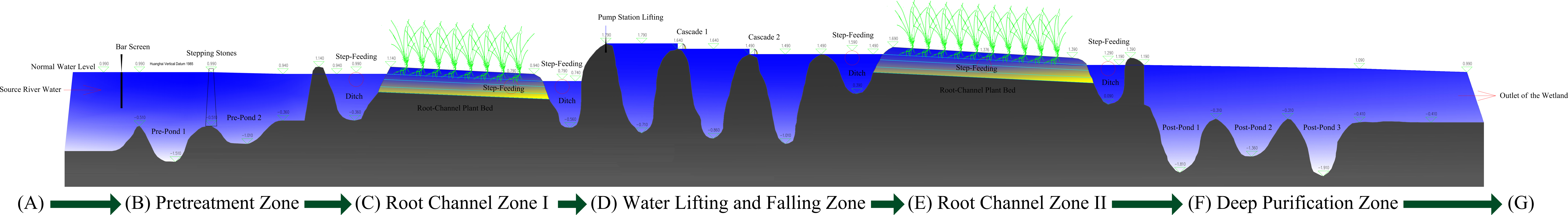

Normal Water Level
Source River Water
Bar Screen
Huanghai Vertical Datum 1985
Stepping Stones
Pre-Pond 1
Pre-Pond 2
Step-Feeding
Ditch
Root-Channel Plant Bed
Step-Feeding
Step-Feeding
Ditch
Pump Station Lifting
Cascade 1
Cascade 2
Step-Feeding
Ditch
Step-Feeding
Root-Channel Plant Bed
Step-Feeding
Ditch
Post-Pond 1
Post-Pond 2
Post-Pond 3
Outlet of the Wetland
(A)
(B) Pretreatment Zone
(C) Root Channel Zone I
(D) Water Lifting and Falling Zone
(E) Root Channel Zone II
(F) Deep Purification Zone
(G)
